# In Vitro Gastrointestinal Digestion and Colonic Catabolism of Mango (*Mangifera indica* L.) Pulp Polyphenols

**DOI:** 10.3390/foods9121836

**Published:** 2020-12-10

**Authors:** José Luis Ordoñez-Díaz, Alicia Moreno-Ortega, Francisco Javier Roldán-Guerra, Victor Ortíz-Somovilla, José Manuel Moreno-Rojas, Gema Pereira-Caro

**Affiliations:** 1 Department of Food Science and Health, Andalusian Institute of Agricultural and Fisheries Research and Training (IFAPA), Alameda del Obispo, Avda. Menéndez-Pidal, s/n, 14004 Córdoba, Spain; josel.ordonez@juntadeandalucia.es (J.L.O.-D.); aliciamorenoortega@hotmail.com (A.M.-O.); franjaviroldan@gmail.com (F.J.R.-G.); victor.ortiz.somovilla@juntadeandalucia.es (V.O.-S.); josem.moreno.rojas@juntadeandalucia.es (J.M.M.-R.); 2 Department of Food Science and Technology, University of Córdoba, Campus Rabanales, Ed. Darwin-anexo, 14071 Córdoba, Spain

**Keywords:** mango polyphenols, simulated in vitro digestion, bioaccessibility, fecal fermentation, degradation products, catabolic pathway

## Abstract

Mango (*Mangifera indica* L.), a fruit with sensorial attractiveness and extraordinary nutritional and phytochemical composition, is one of the most consumed tropical varieties in the world. A growing body of evidence suggests that their bioactive composition differentiates them from other fruits, with mango pulp being an especially rich and diverse source of polyphenols. In this study, mango pulp polyphenols were submitted to in vitro gastrointestinal digestion and colonic fermentation, and aliquots were analyzed by HPLC-HRMS. The main phenolic compounds identified in the mango pulp were hydroxybenzoic acid-hexoside, two mono-galloyl-glucoside isomers and vanillic acid. The release of total polyphenols increased after the in vitro digestion, with an overall bioaccessibility of 206.3%. Specifically, the most bioaccessible mango polyphenols were gallic acid, 3-*O*-methylgallic acid, two hydroxybenzoic acid hexosides, methyl gallate, 3,4-dihydroxybenzoic acid and benzoic acid, which potentially cross the small intestine reaching the colon for fermentation by the resident microbiota. After 48 h of fecal fermentation, the main resultant mango catabolites were pyrogallol, gallic and 3,4-dihydroxybenzoic acids. This highlighted the extensive transformation of mango pulp polyphenols through the gastrointestinal tract and by the resident gut microbiota, with the resultant formation of mainly simple phenolics, which can be considered as biomarkers of the colonic metabolism of mango.

## 1. Introduction

Mango (*Mangifera indica* L.), a fruit with sensorial attractiveness and extraordinary nutritional and phytochemical composition [1], is one of the most consumed tropical varieties in the world [2]. Today, mangoes rank sixth in total production among major fruit crops worldwide contributing over 55 million tons per year to the global fruit market [2], with Europe being one of the main destinations of this global trade (40% of imports). Within Europe, Andalusia is the only area with a significant commercial production of tropical fruits thanks to the presence of a subtropical climate in certain regions. These tropical crops, which include mangoes, avocados and cherimoyas as the main ones, have been cultivated in growing regions such as the coast of Granada and Málaga, allowing this high-quality product to be accessible to the European market within a few hours, thereby eliminated the need for ship and plane transportation, which can take weeks or even months and involve, in many cases, the collection of underripe fruits [3].

Mangoes contain several essential nutrients including carbohydrates, organic acids, dietary fiber and vitamins and minerals. Sugars including sucrose, fructose and glucose are the predominant soluble sugars in mango, while citric acid and malic acid are the major organic acids present in this fruit [4]. Mangoes also contain substantial amounts of non-essential components known as phytochemicals. Bearing all that in mind, growing evidence suggests that the phytochemical composition of mangoes differentiates them from other fruits, with mango pulp an especially rich and diverse source of phenolic compounds, including mangiferin (C2-β-D-glucopyranosyl-1,3,6,7-tetrahydroxyxanthone), flavonoids (quercetin, catechin and epicatechin), phenolic acids (benzoic and gallic), and a wide range of derivatives such as ellagic acid, gallotannins and ellagitannins [5]. The bioactivity of the parent polyphenolics found in mangoes has been widely investigated and found to include antimicrobial [6], anti-inflammatory [7], antidiabetic [8,9] and anti-carcinogenic activities [9,10,11]. Moreover, the health benefits of mangiferin, a singular polyphenol in the mango, has been widely confirmed and continues to attract considerable attention, especially for its potential to reduce the development of degenerative diseases like heart disease and cancer [12,13]. However, little consideration has been given to the impact that human digestion has on bioactivity once a food is consumed. There is now growing evidences that it is not the parent polyphenols of plant foods, rather their metabolites/colonic catabolites that exert health effects in vivo [14]. Moreover, studies into the health benefits of mango have focused mainly on its bark, leaves, peel, and seed/kernel due to their high content of pharmacologically active compounds. In contrast, very little information is available about the flesh/pulp, the part commonly consumed in fresh/processed juices, purees or dried fruits [15]. The polyphenol content of mango pulp is of particular interest given recent clinical observations that sustained consumption of mango pulp (42 days) reduces the systolic blood pressure of lean subjects and maintained the long-term glucose homeostasis in obese subjects. In addition, mango galloyl-derivatives may contribute to the prevention and treatment of obesity and metabolic disorders, benefits that are still to be confirmed [16]. More recently, it has been shown that the consumption of freeze-dried mango pulp can decrease the glucose levels and increase the high-density lipoprotein cholesterol in healthy adults when it is consumed after a high-fat meal [17].

To date, there are two reports describing the in vitro bioaccessibility of mango polyphenols in ‘Ataulfo’ by-products from pulp paste and peel [18] and in mangoes with different ripening stages [19]. More recently, another two studies have shown the importance of colonic microbiota in the metabolism of mango polyphenols by evaluating the bioaccessibility and colonic fermentation of high-dietary fiber mango-based fruit bars [20] or mango bagasse formulations [21]. To further elucidate the bioaccessibility and colonic transformation of mango pulp polyphenols, this study aims to investigate the stability and resultant breakdown products of mango pulp polyphenols during simulated gastrointestinal digestion and in vitro fecal fermentation by ultra-high-performance liquid chromatography coupled to high-resolution mass spectrometry (UHPLC-HRMS).

## 2. Materials and Methods

### 2.1. Chemicals

Magnesium chloride (hexahydrate), calcium chloride as well as sodium chloride were purchased from Fisher Scientific (Madrid, Spain); ammonium carbonate and sodium bicarbonate were acquired by Sigma-Aldrich (Madrid, Spain); and sodium hydrogen carbonate, potassium dihydrogen phosphate, potassium hydrogen phosphate and magnesium sulfate monohydrate were supplier from VWR International Eurolab (Barcelona, Spain). Yeast extracts, peptone, tween 80, hemin, vitamin K, L-cysteine hydrochloride monohydrate, resazurin redox indicator and calcium chloride were acquired from Sigma-Aldrich. The enzymes used in this study were α-amylase (300–1500 U/mg protein) from human saliva, pepsin (3.2–4.5 U/mg protein), porcine pancreatin (4xUPS) and bile salts, all from Sigma-Aldrich. HCl was obtained from Merck (Darmstadt, Germany) and NaOH from Fisher Scientific (Madrid, Spain). Reference standard compounds including benzene-1,2-diol (pyrogallol), 3,4,5-triydroxybenzoic acid (gallic acid), 3,4-dihydroxybenzoic acid, benzoic acid, methylgallate, 4-hydroxy-3-methoxybenzaldehyde (vanillic acid), quercetin, isorhamnetin, 4′-hydroxy-3′-methoxycinnamic acid (ferulic acid), sinapic acid, 3′,4′-dihydroxycinnamic acid (caffeic acid) and mangiferin were purchased from Sigma-Aldrich. 3-*O*-Methylgallic acid, 4-*O*-methylgallic acid, epigallocatechin, epigallocatechin gallate and epicatechin gallate were obtained from Extrasyntheses (France). The acetonitrile and methanol were of LC-MS grade. Note, the nomenclature of the phenolic catabolites used in this paper is based on the recommendation made by Kay et al. [22].

### 2.2. Materials and Sample Preparation

Fresh mangoes (*Mangifera indica* L. cv. Osteen) were kindly supplied by TROP SAT (Málaga, Spain) which certified the mango species. They were washed and the peel manually removed. Then, they were cut into small pieces and milled using a homogenizer (SAMMIC, Madrid, Spain). Afterwards, the mango puree was lyophilized in a freeze dryer ECO EVO (Tred Technology S.R.L., Ripalimosani, Italy) and stored at −80 °C.

### 2.3. In Vitro Gastrointestinal Digestion

The in vitro gastrointestinal digestion procedure was performed according to Moreno-Ortega et al. [23]. The whole process consisted in three steps: the oral, gastric and intestinal phases. Briefly, 2 g of lyophilized sample was firstly submitted to the the oral phase which consisted in 14 mL of a simulated salivary fluid (SSF), 325 µg of α-amylase, 0.1 mL of 0.3 M CaCl_2_ and distilled water up to 20 mL. The oral mixture was incubated at 37 °C in a shaking bath (Unitronic Reciprocating Shaking Bath, model 6,032,011, J.P. Selecta, Barcelona, Spain) during 30 min. Then, the mixture was adjusted to pH 3 and submitted to a gastric phase which consisted in 15 mL of simulated gastric fluids (SGF), 1.19 mL of 0.1 g/mL pepsin solution in 0.1 M HCL, 0.01 mL of 0.3 M CaCl_2_ and distilled water up to a final volume of 40 mL. The gastric mixture was incubated at 37 °C for 120 min. Finally, to carried out the intestinal phase, 22 mL of simulated intestinal fluids (SIF) together with 80 mg of pancreatin, 125 mg of bile salts, 0.08 mL of 0.3M CaCl_2_ and 9.92 mL of distilled water were added. Immediately after, the pH was adjusted to 7 and the whole mixture was incubated at 37 °C during 120 min in a shaking bath. The details of the simulated fluids were described in Moreno-Ortega et al. [23].

Samples were taken before (BOD) and after oral digestion (AOD) and after gastric (AGD) and intestinal digestion (AID) using different experiments. These samples were lyophilized and stored at −80 °C.

### 2.4. In Vitro Colonic Fermentation

The freeze-dried digested mango puree was subjected to in vitro fermentation to simulate the conditions present in the colon following the method described by De Santiago et al. [24] and adapted to our laboratory. Details of the composition of the growth medium are described in De Santiago et al. [24]. The human faecal samples were obtained from three healthy non-smoking volunteers (three males aged between 22 and 34 and with body mass indexes (BMIs) between 20.5 and 25.4). These volunteers had not consumed antibiotics at least 6 months before the study. Forthy eight hours before the faecal sample collection, the volunteers followed a low polyphenol. The samples were collected by the donors in plastic tubes containing an AnaeroGen sachet (Oxoid Ltd., Cambridge, UK) to maintain anaerobic conditions during the transport and were processed within 30 min of passage. The faeces were homogeneized in pre-reduced phosphate buffered saline (PBS). The temperature of the incubation was set to 37 °C using a Unitronic OR circulating water-bath (JP Selecta) and the fermentation bottles were inoculated with homogenized faecal material (10% *w*/*v* of fresh human faeces) for a period of 48 h. After the addition of the lyophilized digested mango (2 g) the bottles were purged with oxygen-free nitrogen (OFN) and sealed airtight and the anaerobiotic conditions were maintained by using a continuous OFN flow. Aliquots (1 mL) of faecal suspensions were taken after 0, 4, 8, 24 and 48 h. The samples were centrifuged at 13,500 rpm at 4 °C for 10 min and stored immediately at −80 °C until analysis.

### 2.5. Extraction of Polyphenols from Digested Mango Samples and from Faecal Incubates

The extraction of polyphenols from in vitro digested mango samples and from faecal samples was adapted from Pereira-Caro et al. [25] and Alañón et al. [26] with some modifications. For the in vitro digested mango samples, 0.25 g of lyophilized sample was homogenized with 1 mL of a methanol/acidified water mixture (80:20, *v*/*v*) with 0.1% formic acid. The samples were centrifuged at 5000 rpm for 10 min at 4 °C, and supernatants were collected. The pellet was re-extracted with 1 mL of the same solvent as described above. All the supernatants were pooled to a final volume of 2 mL. For the fermented mango samples, 0.5 mL of faecal incubates were extracted using 0.5 mL of 0.1% formic acid in methanol/acidified water (80:20, *v*/*v*), vortexed and centrifuged at 5000 rpm for 10 min at 4 °C, and supernatants were collected.

### 2.6. UHPLC-HRMS Polyphenol Analysis

Polyphenols extracted from the in vitro digested mango and faecal incubation samples were analyzed using an Ultimate 3000 RS UHPLC system (Dionex, San José, CA, USA) described previously in Moreno-Ortega et al. [23]. A Zorbax SB-C18 RRHD column (100 × 2.1 mm i.d., 1.8 μm (Agilent, Santa Clara, CA, USA) preceded by a guard precolumn of the same stationary phase and maintained at 40 °C was ued for polyphenol HPLC separation. The flow rate was set to 0.2 mL/min with a 26 min gradient of phase A: deionized water with 0.1% formic acid and B: acetonitrile with 0.1% formic acid. The gradient started at 3% B, was maintained for 2 min, then rose to 65% B in 18 min, before rising to 80% B in 1 min and being maintained for 6 min with a 26 min gradient. Next, the column was equilibrated to the previous conditions within 10 min. The Exactive Orbitrap mass spectrometer, fitted with a heated electrospray ionization probe (ThermoFisher Scientific, San José, CA, USA), operated in negative ionization mode (scanning from 100 to 1000 m/z). The capillary temperature and the heater temperature were set to 300 °C and 150 °C, respectively. 20 units were the sheath gas and the auxiliary gas flow rate, the sweep gas was 3 units, and the spray voltage was 4.00 kV. Xcalibur (3.0 software) was used for data acquisition and data processing.

The polyphenols in digested and fermented mango samples were identified by comparing the exact mass and the retention time with available commercially standards. In the absence of standards, polyphenols were putatively identified by comparing the theoretical exact mass of the molecular ion with its measured accurate mass, and referred to databases or libraries containing HRMS spectral information such as Phenol Explorer (http://phenol-explorer.eu/), Phytohub (http://phytohub.eu/) and Metlin (https://metlin.scripps.edu/landing_page.php?pgcontent=mainPage) databases. Identifications were categorized according to the annotation described by Summer et al. [27] using the MSIMI level. The phenolic compounds were quantified by selecting the theoretical exact mass of the molecular ion by reference to 0.01 to 100 ng/µL standard curves. The linearity was determined for all the commercially available standards. Limits of detection (LOD) and limits of quantification (LOQ) were from 0.01 to 0.2 ng and from 0.05 to 0.8 ng, respectively. In absence of reference compounds, the polyphenols were quantified by reference to the calibration curve of a closely related parent compound.

### 2.7. Bioaccessibility of (Poly)Phenols

The percentage of bioaccessibility (bioaccessibility index) after simulated gastrointestinal digestion [23] was calculated as percentage of the final concentration of each compound after the simulated gastrointestinal digestion and the initial concentration of each compound in the non-digested samples.

### 2.8. Statistical Analysis

Statistical analyses were carried out with three replicate measures of each sample. R software (v. 3.6.3, R Core Team, Vienna, Austria) was used to do the one-way ANOVA to determine significant differences between the different phases of the in vitro gastrointestinal digestion and the fecal fermentation. Then, the Tukey Honestly Significant Difference (HSD) post-hoc test was used for pairwise comparison.

## 3. Results and Discussion

### 3.1. Polyphenols in Mango Pulp Samples

A total of 43 polyphenol compounds belonging to different families were identified in the mango samples. Details of the UHPLC-HRMS characteristics, including the retention time (Rt), the experimental accurate mass and the error (ppm) between the exact accurate mass and the mass found of the detected compounds are summarized in Table 1.

The following compounds were quantified: 16 phenolic acid derivatives, three flavan-3-ol derivatives, five flavanone derivatives, four flavonol derivatives, nine hydroxycinnamic acid derivatives, one xanthone and five gallotannin derivatives (Table 2). The main polyphenols were a hydroxybenzoic acid-hexoside (isomer 2) and two monogalloyl glucoside isomers, followed by the phenolic acid 4-hydroxy-3-methoxybenzaldehyde (vanillic acid), accounting for the 76.2% of the total polyphenols in the samples. Small quantities of other polyphenols were also detected, including pyrogallol (27 µmol/g DW), gallic acid (46 µmol/g DW), gallic acid hexoside (61 µmol/g DW), 3-*O*-methylgallic acid (47 µmol/g DW), 4-*O*-methylgallic acid (69 µmol/g DW), hydroxybenzoic acid hexoside 1 (111 µmol/g DW), syringic acid hexoside (2.7 µmol/g DW), methyl gallate (1 µmol/d DW), methyl digallate ester (isomers 1 and 2) (16 and 1.6 µmol/g DW), two isomers of ferulic acid hexoside (48 and 63 µmol/g DW), three isomers of sinapic acid hexoside (19, 28 and 35 µmol/g DW) and two caffeoyl-hexosides (47 and 3.3 µmol/g DW). Furthermore, the samples presented trace amounts of epicagallocatechin, epigallocatechin gallate, epicatechin gallate, eriodyctiol and two of its hexoside derivatives, hesperetin, quercetin, isorhamnetin and mangiferin. It is evident that the phytochemical composition of mango pulp is very complex, including several polyphenol families, making it difficult to compare their quantities with the literature as the composition is influenced by factors such as cultivar [15,28], storage conditions [29], ripening stage [26,30], and the part of the fruit analyzed, this being one of the factors with the greatest influence on the accumulation of bioactive compounds. For instance, the xanthone mangiferin, the flavonoids quercetin and kaempferol, and the phenolics gallic, ferulic, coumaric and caffeic acids have been shown to be the main polyphenols in mango leaves, peels and mango by-products [20,21] and in ethanolic extracts of peel and pulp residues [31]. In the case of the pulp, the phenolic acids gallic acid, caffeic acid, chlorogenic acid and protocatechuic acid were identified in different mango varieties, with gallic acid being predominant, followed by protocatechuic, chlorogenic, ferulic, vanillic and caffeic acids [32,33,34,35].

### 3.2. Stability and Bioaccessibility of Phenolic Compounds in Mango after Simulated Gastrointestinal Digestion

Table 2 shows the impact of in vitro gastrointestinal digestion on mango polyphenols. After in vitro oral digestion, the total content of polyphenols in the mango significantly increased (from 3.47 to 8.2 mmol/g DW), with a mean recovery of 237.6%. The compounds mostly affected during this oral phase were the phenolic acid derivatives and the galloyl-derivatives.

The phenolic acids significantly increased their concentration from 2169 µmol/g DW to 7026 µmol/g DW, accounting for a mean recovery of 323.9%, mainly due to the substantial increase of individual phenolics including gallic acid (from 46 to 109 µmol/g DW), 4-*O*-methylgallic acid (from 69 to 132 µmol/g DW), the two hydroxybenzoic acid hexoside isomers (from 1370 to 6078 µmol/g DW), methyl gallate (from 1 to 28 µmol/g DW) and the two methyl-gallate ester isomers (from 17.6 to 32.4 µmol/g DW). Meanwhile the galloyl-derivatives presented a slight decrease in concentration from 1010 to 923 µmol/g DW, showing a recovery of 91.3% after the oral phase, likely due to the decrease in the mono-galloyl glucoside isomers.

The remaining compounds in the mango, belonging to flavan-3-ol, flavanones, hydroxycinnamic acid and xanthone families, were stable under the oral digestion conditions, presenting mean recovery rates between 95.2% for flavonol derivatives and 116.7% for mangiferin. The increase in gallic acid and hydroxybenzoic acid hexoside during the oral phase is likely due to enzymatic activity and the pH, which could induce the breakdown of these compounds from other food constituents, as previously reported by other authors in soy milk [36] and persimmon fruits [37].

After in vitro gastric digestion, the total content of polyphenols in mango pulp significantly decreased compared to those obtained after oral digestion (from 8.2 to 6.07 mmol/g DW), but the total concentration was still higher than the initial quantities of polyphenols in the mango pulp. Despite the notable decrease in the polyphenol content after gastric digestion of almost all the polyphenol classes—losses ranging from 100% for hesperetin glucosides isomers to 20% for monogalloyl-glucoside—there were some specific compounds that presented significant increases in their concentration under the gastric conditions. Specifically, as likely occurred during the oral phase, the phenolic acids, namely gallic acid, the two hydroxybenzoic acid hexoside isomers, methyl gallate and the two methyl-gallate ester isomers, were positively influenced by the gastric conditions and substantially increased their concentrations after in vitro gastric digestion. Recovery rates ranged between 156.3 and 2900%, while the two tetra-galloyl glucoside isomers presented recoveries of 333.3 and 157%, respectively. These changes can be explained taking into consideration that during the gastric phase, the polyphenols, which are hydrogen bonding structures or are covalently linked to cell wall polysaccharides in the food matrix, can be released due to the low pH and pepsin activity [38]. In keeping with our results, Lucas-González et al. [37] found a significant increase in gallic acid content in persimmon fruits after the gastric phase.

After the in vitro intestinal digestion, the total polyphenol content in the mango pulp continue increasing, from 6.07 to 7.15 mmol/g DW, with a mean bioaccessibility index of 206.3%. This increase was mostly due to the huge increase in the concentration of specific phenolic compounds after the intestinal digestion, such as gallic acid (from 188 to 267 µmol/g DW), 3-*O*-methylgallic acid (from 85 to 2259 µmol/g DW), methyl gallate (from 29 to 737 µmol/g DW), in addition to the appearance of 3,4-dihydroxybenzoic acid (176 µmol/g DW) and benzoic acid (84 µmol/g DW), arguably from the hydrolysis of monogallyl glucoside and hydroxybenzoic acid hexoside, respectively. At intestinal level, factors such as pH and the action of the enzymes (pancreatin) and bile salts could favor the breakage of the weak bond between the supramolecular structures of the food matrix and the polyphenols, mainly the ones with low molecular weight, releasing them from the food matrix during the digestion and, therefore, increasing their bioaccessibility [21]. All these compounds are potentially available for absorption at the end of intestinal digestion. Moreover, despite the increase in the total quantities of polyphenols after in vitro digestion, the remaining polyphenols, including the flavan-3-ols, flavanols, hydroxycinnamic acid derivatives and xanthone, were negatively affected by the digestive process and their concentration significantly decreased during the digestive process. The losses ranged from 6.2% for methyl digallate ester 1 to completely disappearing, as in the case of 4-*O*-methylgallic acid, methyl-digallate ester (isomer 2), epigallocatechin gallate, epicatechin gallate, eriodictyol hexoside 1 and 2, hesperetin-glucoside (1 and 2), quercetin hexoside 1, isorhamnetin hexoside 1 and caffeoyl-quinic acid 1. Overall, the most bioaccessible compounds of the mango pulp were gallic acid, 3-*O*-methylgallic acid, the two hydroxybenzoic acid hexosides, methyl gallate, 3,4-dihydroxybenzoic acid and benzoic acid, which potentially cross the small intestine and reach the colon for fermentation by the resident microbiota. These results are in keeping with those reported by Blancas-Benitez et al. [18], who also found that the major polyphenols released during the in vitro digestion process of mango paste were gallic acid and hydroxybenzoic acids.

### 3.3. Degradation of Mango Polyphenols during Fecal Fermentation

After the in vitro digestion process, which mimics the steps that would occur in vivo before the food enters the large intestine to be fermented, the digested mango pulp samples were incubated with a fresh and homogenized fecal suspension from three donors for a period of 48 h under anaerobic conditions and analyzed by HPLC-HRMS. Details of the UHPLC-HRMS characteristics of the detected microbial-derived compounds are shown in Table 3.

The results from the in vitro fecal fermentation, presented in Table 4, revealed that the human microbiota gradually converted the mango polyphenols remaining after the in vitro gastrointestinal digestion. The overall quantities of mango polyphenols and their catabolites present after 4, 8, 24 and 48 h of fermentation were 4095, 4202, 2286 and 2043 µmol/g DW, respectively, corresponding to 101, 103, 56 and 50% of the initial amounts (Table 4). The major end products were pyrogallol, gallic acid and 3,4-dihydroxybenzoic acid, comprising 97.4% of the total catabolites; the remaining 2.5% comprised the minor catabolites such as hydroxybenzoic acid hexoside 2, 3-(4′-dihydroxy-phenyl)propanoic acid, 3-phenylacetic acid, (-)-epicatechin, eriodictyol, caffeoyl hexoside 1 and the monogalloyl hexoside 1 (Table 4).

**Figure 1:**
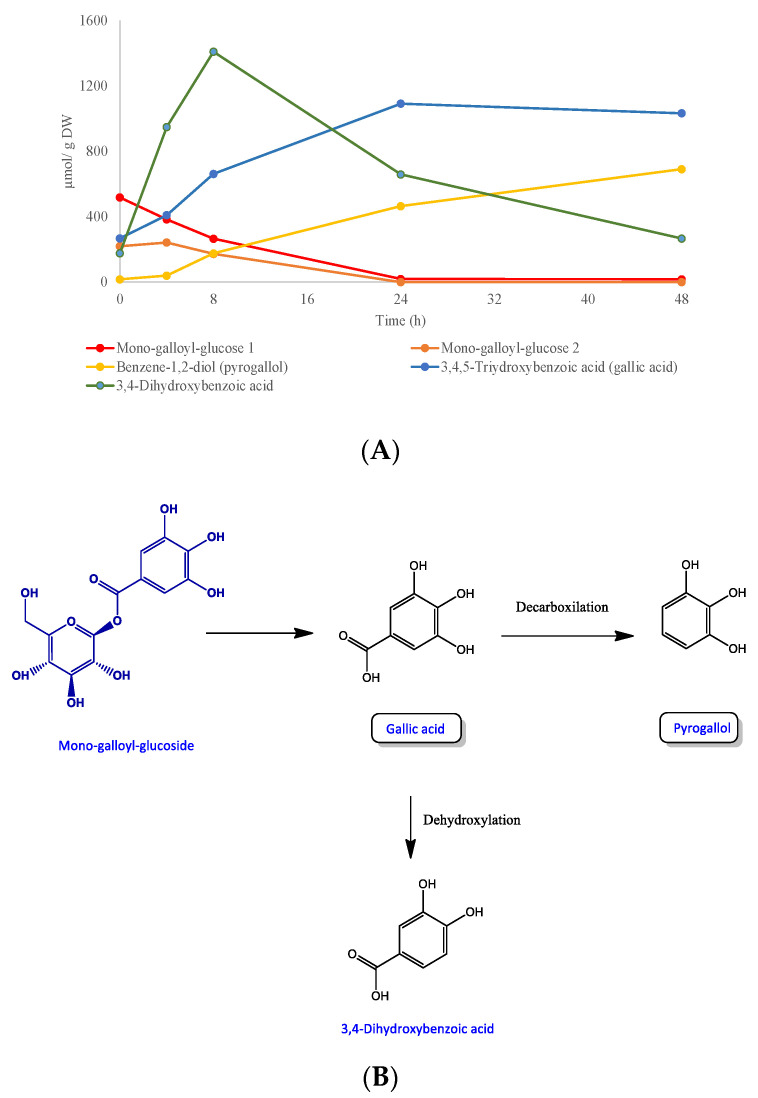
(**A**) Monogalloyl hexoside degradation profiles over 48 h of incubation with human faeces. Data expressed as µmol ± SD (*n* = 3). (**B**) Proposed catabolic pathway for the colonic transformation of mono-galloyl-hexoside in humans. Framed names indicated major catabolites.

After an incubation time of 4 h, significant quantities of pyrogallol (40 µmol/g DW), gallic acid (410 µmol/g DW) and 3,4-dihydroxybenzoic acid (949 µmol/g DW) were detected, attaining the highest accumulation of these compounds after incubation periods of 48, 24 and 8 h, respectively (176–691 µmol/g DW, 662–1033 µmol/g DW and 1410–267 µmol/g DW). A concomitant decrease to almost zero of the mono-galloyl-glucosides and hydroxybenzoic acid-hexosides was also evidenced after 48 h of incubation (Table 4, Figure 1A). These data indicate that a plausible mechanism for galloyl-glucoside degradation by human microbiota is, first, the microbial-mediated transformation of mono-galloyl-glucoside into gallic acid, as the first catabolic event, which is further decarboxylated or dehydroxylated producing pyrogallol and 3,4-dihydroxybenzoic acid, as proposed in Figure 1B. In line with our results, Hernandez-Maldonado et al. [20] reported that gallic acid was identified during the in vitro fermentation of digested mango bars, the maximum amount being reached after 6 h of incubation. Moreover, gallic acid has been identified in human urine after mango pulp consumption [39,40] and was inversely correlated with the urinary excretion of pyrogallol, indicating the colonic origin of this catabolite.

The compound (-)-epicatechin was initially detected after 4 h and 8 h of incubation, respectively (8.2 and 0.6 µmol/g DW). The appearance of this compound was marked by a gradual decrease in epigallocatechin, which disappeared completely after 24 h of incubation (Table 4). Epicatechin can be further transformed into 5-(hydroxyphenyl)-γ-valerolactones and phenylhydroxyvaleric acid derivatives by dihydroxylation and ring fission transformations [41], but none of these catabolites were detected in our study.

**Figure 2:**
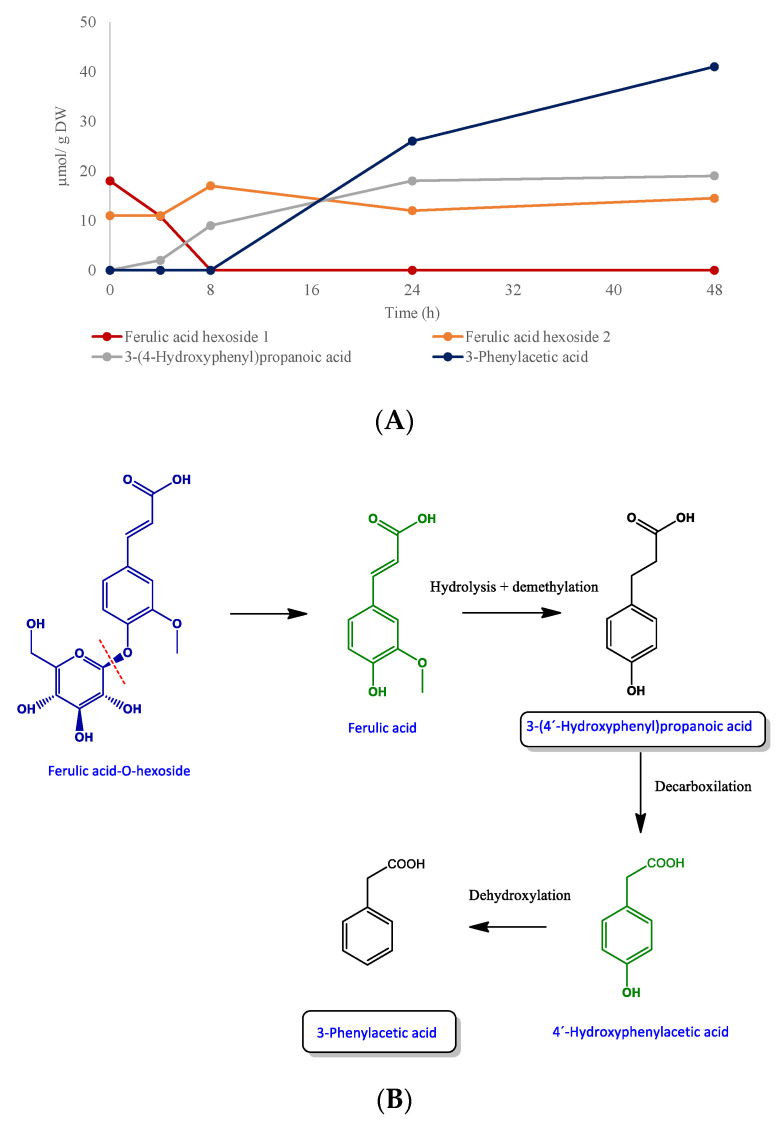
(**A**) Ferulic acid-hexoside degradation profiles over 48 h of incubation with human faeces. Data expressed as µmol ± SD (*n* = 3). (**B**) Proposed catabolic pathway for the colonic transformation of ferulic acid-hexoside in humans. Black: detected metabolites; green; non-detected metabolites. Framed names indicate major catabolites.

Small quantities of other polyphenols such as syringic acid glucoside 1 and 2, vanillic acid, sinapic acid-glucoside and caffeoyl-hexoside 1 and 2 found in digested mango samples before fermentation disappeared during the faecal incubation. In contrast, low levels of 3-(4-hydroxyphenyl)propanoic acid (2 µmol/g DW) and 3-phenylacetic acid (26 µmol/g DW) were detected after 4 and 24 h respectively of incubation of the digested mango samples with faecal material (Table 4). These levels, increased thereafter, reaching maximum amounts after 48 h of incubation (19 and 41 µmol/g DW, respectively) (Figure 2A).

These catabolites were arguably formed by the degradation of ferulic acid-hexoside via hydrolysis and the demethylation of ferulic acid to 3-(4-hydroxyphenyl)propanoic acid, and further decarboxylation and dihydroxylation to 3-phenylacetic acid following the procedure described in Figure 2B. Indeed, these catabolites have been reported to be faecal fermentation products of ferulic acid [25].

**Figure 3:**
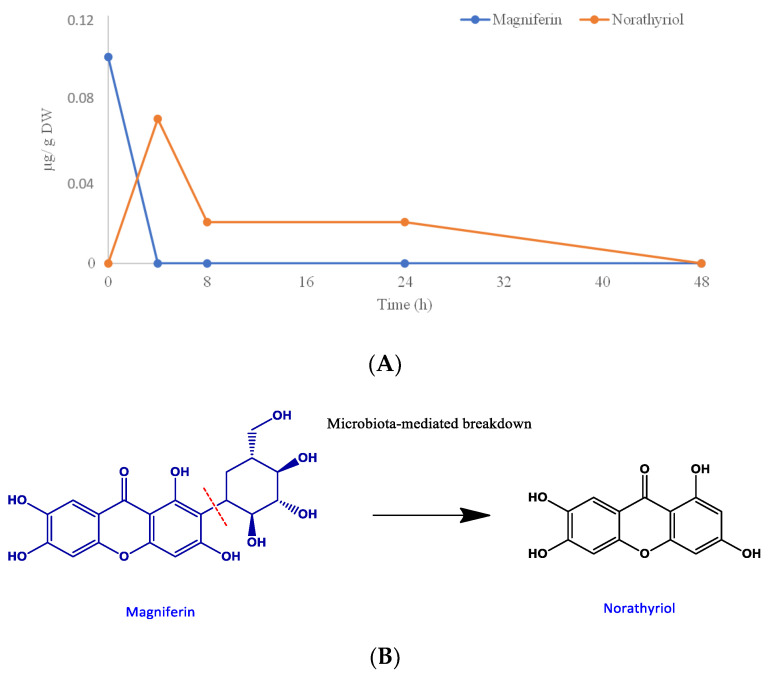
(**A**) Mangiferin degradation profiles over 48 h of incubation with human faeces. Data expressed as µmol ± SD (*n* = 3). (**B**) Proposed catabolic pathway for the colonic transformation of mangiferin in humans.

The incubation of faecal slurries with the digested mango polyphenols also resulted in the appearance of norathyriol after 4 h of incubation (0.07 µmol/g DW), the levels decreasing slightly during the fermentation process until disappearing after 48 h of incubation (Table 4, Figure 3A). This compound has been described as the main microbial-degradation product of mangiferin, in which faecal bacteria act by cleaving the C-glycosyl bond leading to aglycone [42,43] via the pathway illustrated in Figure 3B. The Bacteroides species named MANG, found in human faeces, is reported to be responsible for this transformation [44]. In view of these results, the catabolite norathyriol can be proposed as a biomarker of the colonic degradation of mangiferin.

Overall, the information from this in vitro study is of value because pointed out the great transformation of mango polyphenols through the gastrointestinal tract as well as by the colonic microbiota. The derived catabolites identified in our study merits further investigation to elucidate the potential protective effects of mango consumption in vivo. Therefore, these phenolic catabolites will be the focus to the design of ex vivo cell-based experiments (next step) aimed at elucidating the underlying modes of action of the protective effect of mango polyphenol on health.

Moreover, future research will involve the evaluation of the bioavailability of mango pulp polyphenols in humans. The information from the in vivo human studies together with those from this in vitro study would provide a detailed evaluation of the fate of mango pulp polyphenols through the body. Furthermore, this will help to identify additionally potential mango bioactive compounds.

## 4. Conclusions

The present study investigated the effects of in vitro simulated gastrointestinal digestion and colonic fermentation on the stability and bioaccessibility of mango pulp polyphenols, and evaluated the resultant breakdown products after faecal fermentation by the resident human microbiota. During in vitro gastrointestinal digestion of mango pulp polyphenols there was a significant increase in total polyphenols, with the phenols gallic acid, 3-*O*-methylgallic acid, methyl gallate, 3,4-dihydroxybenzoic acid and benzoic acid being the most accessible. These are arguably the result of the decrease in other compounds such as monogallyl glucoside and hydroxybenzoic acid hexoside. At colonic level, they were subjected to the action of the microbiota, being converted principally to gallic acid, pyrogallol and 3,4-dihydroxybenoic acid, compounds potentially involved in the health benefits of mango pulp consumption.

## Figures and Tables

**Table 1:** UHPLC-HRMS Characteristics of Polyphenol Compounds Identified and Quantified in Mango Samples. RT: retention time; [M-H]^−^ Exp: experimental exact mass; Δ: mass error.

RT (min)	Compound	Chemical Structure	[M-H]^−^ Exp. (m/z)^−^	Δ (ppm)	MSIMI ^a^
	*Phenolic Acid Derivatives*				
1.3	Benzene-1,2-diol (pyrogallol)	C_6_H_6_O_3_	125.0233	0.39	1
3.1	3,4,5-Triydroxybenzoic acid (gallic acid)	C_7_H_6_O_5_	169.0137	0.77	1
6.6	Gallic acid hexoside	C_13_H_16_O_11_	347.0608	1.73	2
8.8	3-*O*-Methylgallic acid	C_8_H_7_O_5_	183.0287	1.8	1
11.9	4-*O*-Methylgallic acid	C_8_H_7_O_5_	183.0287	1.8	1
8.7	3,4-Dihydroxybenzoic acid	C_7_H_6_O_4_	153.0182	0.23	1
9	Benzoic acid	C_7_H_6_O_2_	121.0284	0.74	1
4.7	Hydroxybenzoic acid hexoside 1	C_13_H_16_O_8_	299.0767	3.76	2
7	Hydroxybenzoic acid hexoside 2	C_13_H_16_O_8_	299.0767	3.76	2
7.4	Syringic acid glucoside 1	C_15_H_20_O_10_	359.0972	1.91	2
7.8	Syringic acid glucoside 2	C_15_H_20_O_10_	359.0972	1.91	2
8.8	Methyl gallate	C_8_H_8_O_5_	183.0287	1.64	2
11.9	Methyl digallate ester 1	C_15_H_21_O_9_	335.0397	4.3	2
13	Methyl digallate ester 2	C_15_H_21_O_9_	335.0397	4.3	2
7.1	4-Hydroxy-3-methoxybenzaldehyde (vanillic acid)	C_8_H_8_O_4_	167.0344	058	1
3.8	Galloyl-quinic acid	C_14_H_16_O_10_	343.0659	1.63	2
	*Flavan-3-ol derivatives*				
8.4	Epigallocatechin	C_15_H_14_O_7_	305.0655	2.39	1
9.8	Epigallocatechin gallate	C_22_H_18_O_11_	457.0765	3.87	1
11.1	Epicatechin gallate	C_22_H_18_O_11_	457.0765	3.87	1
	*Flavanone Derivatives*				
8.8	Eriodyctiol	C_15_H_12_O_6_	287.0550	3.22	1
9.7	Eriodyctiol hexoside 1	C_21_H_22_O_11_	449.1078	1.59	2
10.4	Eriodyctiol hexoside 2	C_21_H_22_O_11_	449.1078	1.59	2
9.7	Hesperetin glucoside 1	C_22_H_24_O_11_	463.1234	0.18	2
12.3	Hesperetin glucoside 2	C_22_H_24_O_11_	463.1234	0.18	2
	*Flavonol Derivatives*				
8.3	Quercetin-hexoside 1	C_21_H_20_O_12_	463.0877	2.09	2
11.1	Quercetin-hexoside 2	C_21_H_20_O_12_	463.0877	2.09	2
9.5	Isorhamnetin hexoside 1	C_22_H_22_O_12_	477.1027	2.05	2
9.9	Isorhamnetin hexoside 2	C_22_H_22_O_12_	477.1027	2.05	2
	*Hydroxycinnamic acid derivatives*				
8.6	Ferulic acid hexoside 1	C_16_H_20_O_9_	355.1023	2.31	2
9.5	Ferulic acid hexoside 2	C_16_H_20_O_9_	355.1023	2.31	2
9	Sinapic acid hexoside 1	C_17_H_22_O_10_	385.1129	2.58	2
9.3	Sinapic acid hexoside 2	C_17_H_22_O_10_	385.1129	2.58	2
9.6	sinapic acid hexoside 3	C_17_H_22_O_10_	385.1129	2.58	2
8	Caffeoyl-hexoside 1	C_15_H_18_O_9_	341.0867	2.53	2
8.7	Caffeoyl-hexoside 2	C_15_H_18_O_9_	341.0867	2.53	2
8.9	Caffeoyl-quinic 1	C_16_H_18_O_9_	353.0867	1.96	2
10	Caffeoyl-quinic 2	C_16_H_18_O_9_	353.0867	1.96	2
	*Xanthone*				
9.4	Mangiferin	C_19_H_18_O_11_	421.0765	1.52	1
	*Galloyl derivatives*				
2.7	Mono-galloyl-glucose 1	C_13_H_16_O_10_	331.0665	3.61	2
6.6	Mono-galloyl-glucose 2	C_13_H_16_O_10_	331.0665	3.61	2
9.9	Tetra-*O*-galloyl glucoside 1	C_34_H_28_O_19_	787.0994	2.04	2
10.7	Tetra-*O*-galloyl glucoside 2	C_34_H_28_O_19_	787.0994	2.04	2
11.3	Penta-*O*-galloyl glucoside	C_41_H_32_O_26_	939.1035	1.87	2

^a^ Metabolite Standards Initiative Metabolite Identification (MSIMI) levels [26]. Reference compounds were available for all compounds identified at MSIMI level 1.

**Table 2:** Polyphenol compounds in mango puree samples before and after in vitro gastrointestinal digestion. Results are expressed as mean ± standard deviation (µmol/g DW) (*n* = 3). BOD: before oral digestion; AOD: after oral digestion; AGD: after gastric digestion; AID: after intestinal digestion. % Rem: % remained; BI: Bioaccessibility Index.

Compounds	BOD	AOD	% Rem	AGD	% Rem	AID	*BI*	*p*-Value
*Phenolic acid derivatives*								
Benzene-1,2-diol (pyrogallol)	27 ± 3	18 ± 3	66.7	21 ± 9	77.8	17 ± 4	63	ns
3,4,5-Trihydroxybenzoic acid (gallic acid)	46 ± 4 ^d^	109 ± 16 ^c^	237	188 ± 26 ^b^	408.7	267 ± 36 ^a^	580.4	***
Gallic acid hexoside	61 ± 3 ^a^	49 ± 9 ^b^	80.3	42 ± 6 ^b^	68.9	22 ± 3 ^c^	36.1	***
3-*O*-Methylgallic acid	47 ± 4 ^b^	86 ± 8 ^b^	183	85 ± 13 ^b^	180.9	2259 ± 406 ^a^	4806	***
4-*O*-Methylgallic acid	69 ± 7 ^c^	132 ± 28 ^a^	191.3	112 ± 18 ^b^	162.3	n.d. ^d^	-	***
3,4-Dihydroxybenzoic acid	n.d.	n.d.		n.d.		176 ± 39 ^a^	100	***
Benzoic acid	n.d.	n.d.		n.d.		84 ± 15 ^a^	100	***
Hydroxybenzoic acid hexoside 1	111 ± 45 ^c^	434 ± 88 ^ab^	391	472 ± 141 ^a^	425.2	322 ± 110 ^b^	290.1	***
Hydroxybenzoic acid hexoside 2	1259 ± 327 ^d^	5644 ± 571 ^a^	448.3	3985 ± 444 ^b^	316.5	2287 ± 208 ^c^	181.7	***
Syringic acid glucoside 1	2.7 ± 0.0 ^a^	2.7 ± 0.3 ^a^	100	1.7 ± 0.3 ^b^	63	0.8 ± 0.1 ^c^	29.6	***
Syringic acid glucoside 2	1.2 ± 0.1 ^a^	1.1 ± 0.1 ^b^	91.7	0.5 ± 0.1 ^c^	41.7	0.1 ± 0.0 ^d^	8.3	***
Methyl gallate	1 ± 1 ^b^	28 ± 3 ^b^	2800	29 ± 4 ^b^	2900	737 ± 132 ^a^	737	***
Methyl digallate ester 1	16 ± 1 ^b^	30 ± 6 ^a^	187.5	26 ± 5 ^ab^	162.5	15 ± 13 ^b^	93.8	*
Methyl digallate ester 2	1.6 ± 0.2 ^b^	2.4 ± 0.7 ^a^	150	2.5 ± 0.5 ^a^	156.3	n.d. ^c^	-	***
4-Hydroxy-3-methoxybenzaldehyde (vanillic acid)	522 ± 15 ^a^	486 ± 60 ^a^	93.1	372 ± 71 ^b^	7.3	158 ± 48 ^c^	30.3	***
Galloyl-quinic acid	4.4 ± 0.1 ^a^	3.5 ± 0.4 ^b^	79.5	3.3 ± 0.4 ^b^	75	1.7 ± 0.3 ^c^	38.6	***
*Total Phenolic Acid Derivatives*	2169 ± 410 ^c^	7026 ± 793^a^	323.9	5152 ± 738 ^b^	246.2	6347 ± 1014 ^a^	292.6	**
*Flavan-3-ol derivatives*								
Epigallocatechin	37 ± 0.5 ^a^	38 ± 3 ^a^	102.7	24 ± 2 ^b^	64.9	18 ± 2 ^c^	48.6	***
Epigallocatechin gallate	4.3 ± 0.2 ^a^	4.4 ± 1.4 ^a^	102.3	0.9 ± 0.2 ^b^	20.9	0.00 ± 0.00 ^b^	-	***
Epicatechin gallate	0.21 ± 0.02 ^b^	0.27 ± 0.04 ^a^	128.6	0.02 ± 0.01 ^c^	9.5	0.00 ± 0.00 ^c^	-	***
*Total Flavan-3-ol derivatives*	41.5 ± 0.7 ^a^	42.7 ± 4.4 ^a^	102.8	24.9 ± 2.2 ^b^	60	18 ± 2 ^c^	43.4	***
*Flavanones Derivatives*								
Eriodyctiol	0.66 ± 0.04 ^a^	0.68 ± 0.09 ^a^	103	0.27 ± 0.05 ^b^	40.9	n.d. ^c^	-	***
Eriodyctiol hexoside 1	0.11 ± 0.01 ^a^	0.09 ± 0.03 ^a^	81.8	0.03 ± 0.01 ^b^	27.3	n.d. ^c^	-	***
Eriodyctiol hexoside 2	0.04 ± 0.02 ^ab^	0.05 ± 0.02 ^a^	125	0.02 ± 0.01 ^b^	50	n.d. ^c^	-	***
Hesperetin glucoside 1	0.12 ± 0.05 ^a^	0.17 ± 0.07 ^a^	141.7	n.d. ^b^	0.0	n.d. ^b^	-	***
Hesperetin glucoside 2	0.03 ± 0.00 ^a^	n.d. ^b^	0	n.d.^b^	0.0	n.d. ^b^	-	***
*Total Flavanones Derivatives*	0.96 ± 0.12 ^a^	0.99 ± 0.21 ^a^	103.1	0.32 ± 0.07 ^b^	33.3	n.d. ^c^	-	***
*Flavonols Derivatives*								
Quercetin-hexoside 1	0.03 ± 0.01 ^a^	0.02 ± 0.01 ^b^	66.7	0.02 ± 0.01 ^b^	66.7	n.d. ^c^	-	**
Quercetin-hexoside 2	0.10 ± 0.00 ^a^	0.10 ± 0.01 ^a^	100	0.06 ± 0.01 ^b^	60	0.02 ± 0.01 ^c^	20	***
Isorhamnetin hexoside 1	0.01 ± 0.00 ^a^	0.01 ± 0.00 ^a^	100	n.d. ^b^	0	n.d. ^b^	-	**
Isorhamnetin hexoside 2	0.70 ± 0.01 ^a^	0.67 ± 0.07 ^a^	95.7	0.50 ± 0.07 ^b^	71.4	0.37 ± 0.07 ^c^	52.9	***
*Total flavonol derivatives*	0.84 ± 0.02 ^a^	0.80 ± 0.09 ^a^	95.2	0.58 ± 0.10 ^b^	69	0.39 ± 0.08 ^c^	46.4	**
*Hydroxycinnamic acid derivatives*								
Ferulic acid hexoside 1	48 ± 2 ^a^	48 ± 4 ^a^	100	27 ± 3 ^b^	56.3	20 ± 3 ^c^	41.7	***
Ferulic acid hexoside 2	63 ± 3 ^a^	70 ± 8 ^a^	111.1	37 ± 3 ^b^	58.7	13 ± 2 ^c^	20.6	***
Sinapic acid hexoside 1	19 ± 2 ^a^	20 ± 1 ^a^	105.3	11 ± 1 ^b^	57.9	7 ± 2 ^c^	36.8	***
Sinapic acid hexoside 2	28 ± 2 ^a^	31 ± 4 ^a^	110.7	20 ± 3 ^b^	71.4	11 ± 1 ^c^	39.3	***
sinapic acid hexoside 3	35 ± 2 ^a^	35 ± 4 ^a^	100	19 ± 2 ^b^	54.3	8 ± 2 ^c^	22.0	***
Caffeoyl-hexoside 1	47 ± 2 ^a^	40 ± 4 ^b^	85.1	17 ± 3 ^c^	36.2	7.9 ± 0.8 ^d^	16.8	***
Caffeoyl-hexoside 2	3.3 ± 0.6 ^a^	3.8 ± 0.9 ^a^	115.2	1.6 ± 0.3 ^b^	48.5	0.6 ± 0.3 ^c^	18.2	***
Caffeoyl-quinic 1	0.5 ± 0.3 ^a^	0.4 ± 0.1 ^a^	80	n.d. ^b^	0	n.d. ^b^	-	***
Caffeoyl-quinic 2	2.3 ± 0.3 ^a^	2.5 ± 0.2 ^a^	108.7	1.09 ± 0.19 ^b^	47.4	0.02 ± 0.05 ^c^	-	***
*Total Hydroxycinnamic acid derivatives*	246.0 ± 14.2 ^a^	251 ± 26 ^a^	101.9	133.7 ± 15.5 ^b^	54.3	67.5 ± 11.1 ^c^	27.4	***
*Xanthone*								
Mangiferin	0.12 ± 0.01 ^ab^	0.14 ± 0.03 ^a^	116.7	0.09 ± 0.02 ^b^	75	0.10 ± 0.02 ^b^	83.3	**
*Gallotannins derivatives*								
Mono-galloyl-glucose	481 ± 16 ^a^	453 ± 30 ^a^	94.2	385 ± 30 ^b^	80.0	516 ± 85 ^a^	107.3	**
Mono-galloyl-glucose	526 ± 34 ^a^	465 ± 53 ^b^	88.4	373 ± 49 ^c^	70.9	197 ± 21 ^d^	37.5	***
Tetra-*O*-galloyl glucoside	0.15 ± 0.01 ^c^	0.33 ± 0.06 ^b^	220	0.5 ± 0.2 ^a^	333.3	0.37 ± 0.09 ^b^	246.7	***
Tetra-*O*-galloyl glucoside	1.72 ± 0.08 ^c^	2.3 ± 0.3 ^b^	133.7	2.7 ± 0.4 ^a^	157	2.2 ± 0.4 ^bc^	127.9	*
Penta-*O*-galloyl glucoside	1.9 ± 0.6 ^b^	2.0 ± 0.7 ^b^	107.9	2.0 ± 0.4 ^b^	105.3	8 ± 2 ^a^	421.1	***
*Total Galloyl derivatives*	1011 ± 51 ^a^	923 ± 84 ^b^	91.3	763 ± 80 ^c^	75.5	724 ± 108 ^c^	71.6	**
TOTAL POLYPHENOLS	3469 ± 476 ^d^	8244 ± 908 ^ab^	237.6	6075 ± 836 ^c^	180.5	7156 ± 1135 ^bc^	206.3	**

Different letters within each row indicate significant difference among samples Ns: non-significant; * *p*-value < 0.05; ** *p*-value < 0.01; *** *p*-value < 0.001. n.d.: not detected.

**Table 3:** UHPLC-HRMS Characteristics of Mango Polyphenol Catabolites Identified after fecal fermentation.

RT (min)	Catabolites	Chemical Structure	[M-H]^−^ Exp. (m/z)	Δ (ppm)	MSIMI ^a^
10.6	3-(4-Hydroxyphenyl)propanoic acid	C_9_H_10_O_3_	165.0546	1.54	1
7.9	3-Phenylacetic acid	C_8_H_8_O_2_	135.0440	0.84	1
8.8	(-)-Epicatechin	C_15_H_14_O_6_	289.0706	0.14	1
10.4	Norathyriol	C_13_H_8_O_6_	259.0216	−2.07	2

^a^ Metabolite Standards Initiative Metabolite Identification (MSIMI) levels [27]. Reference compounds were available for all compounds identified at MSIMI level 1.

**Table 4:** Stability of native mango polyphenols and catabolites produced during 0, 4, 8, 24 and 48 h of faecal fermentation. Results are expressed as mean ± standard deviation (µmol/g DW) (*n* = 3).

Compounds	0 h		4 h		8 h		24 h		48 h		*p*-Value
	Control	Mango	Control	Mango	Control	Mango	Control	Mango	Control	Mango	
Phenolic acid derivatives											
Benzene-1,2-diol (pyrogallol)	n.d. ^f^	17 ± 4 ^e^	n.d. ^f^	40 ± 2 ^d^	n.d. ^f^	176 ± 5 ^c^	n.d. ^f^	465 ± 10 ^b^	n.d. ^f^	691 ± 49 ^a^	***
3,4,5-Triydroxybenzoic acid (gallic acid)	n.d. ^e^	268 ± 36 ^d^	n.d. ^e^	410 ± 11 ^c^	n.d. ^e^	662 ± 61 ^b^	n.d. ^e^	1092 ± 61 ^a^	n.d. ^e^	1033 ± 38 ^a^	***
Gallic acid hexoside	n.d. ^c^	21.9 ± 0.9 ^a^	n.d. ^c^	1.8 ± 0.1 ^b^	n.d. ^c^	1.1 ± 0.1 ^b^	n.d. ^c^	n.d. ^c^	n.d. ^c^	n.d. ^c^	***
3,4-Dihydroxybenzoic acid	n.d. ^f^	177 ± 39 ^e^	n.d. ^f^	949 ± 108 ^b^	n.d. ^f^	1410 ± 85 ^a^	n.d. ^f^	659 ± 184 ^c^	n.d. ^f^	267 ± 28 ^d^	***
Hydroxybenzoic acid hexoside 1	n.d. ^c^	323 ± 56 ^a^	n.d. ^c^	338 ± 5 ^a^	n.d. ^c^	217 ± 52 ^b^	n.d. ^c^	n.d. ^c^	n.d. ^c^	n.d. ^c^	***
Hydroxybenzoic acid hexoside 2	n.d. ^d^	2294 ± 102 ^a^	n.d. ^d^	1569 ± 36 ^b^	n.d. ^d^	1080 ± 68 ^c^	n.d. ^d^	41 ± 13 ^d^	n.d. ^d^	27 ± 2 ^d^	***
Syringic acid glucoside 1	n.d. ^c^	0.9 ± 0.1 ^a^	n.d. ^c^	0.7 ± 0.0 ^b^	n.d. ^c^	0.7 ± 0.1 ^b^	n.d. ^c^	n.d. ^c^	n.d. ^c^	n.d. ^c^	***
Syringic acid glucoside 2	n.d. ^b^	0.2 ± 0.0 ^a^	n.d. ^b^	0.1 ± 0.0 ^a^	n.d. ^b^	n.d. ^b^	n.d. ^b^	n.d. ^b^	n.d. ^b^	n.d. ^b^	***
4-Hydroxy-3-methoxybenzaldehyde (vanillic acid)	n.d. ^c^	162 ± 14 ^a^	n.d. ^c^	104 ± 44 ^b^	n.d. ^c^	167 ± 12 ^a^	n.d. ^c^	n.d. ^c^	n.d. ^c^	n.d. ^c^	***
3-(4-Hydroxyphenyl)propanoic acid	n.d. ^d^	n.d. ^d^	0.9 ± 0.1 ^d^	2 ± 1 ^c^	0.4 ± 0.2 ^c^	9 ± 1 ^b^	0.4 ± 0.1 ^d^	18 ± 3 ^a^	n.d. ^e^	19 ± 1 ^a^	***
3-Phenylacetic acid	n.d. ^b^	n.d. ^b^	n.d. ^b^	n.d. ^b^	n.d. ^b^	n.d. ^b^	n.d. ^b^	26 ± 11 ^a^	n.d. ^b^	41 ± 8 ^a^	***
Flavan-3-ol derivatives											
Epigallocatechin	n.d. ^f^	21 ± 1 ^a^	n.d. ^f^	13 ± 1 ^b^	n.d. ^f^	5.8 ± 0.8 ^c^	n.d. ^f^	1.3 ± 0.1 ^e^	n.d. ^f^	1.5 ± 0.1 ^e^	***
(-)-Epicatechin	n.d. ^d^	n.d. ^d^	n.d. ^d^	8.2 ± 0.3 ^a^	n.d. ^d^	7.5 ± 0.0 ^a^	n.d. ^d^	5.7 ± 0.0 ^b^	n.d. ^d^	2.7 ± 0.2 ^c^	***
Flavanone derivatives											
Eriodyctiol	n.d. ^c^	n.d. ^c^	n.d. ^c^	n.d. ^c^	n.d. ^c^	0.4 ± 0.1 ^c^	n.d. ^c^	1.6 ± 0.4 ^b^	n.d. ^c^	2.3 ± 0.3 ^a^	***
Hydroxycinnamic acid derivatives											
Ferulic acid hexoside 1	n.d. ^c^	18 ± 2 ^a^	n.d. ^c^	10.9 ± 0.8 ^b^	n.d. ^c^	n.d. ^c^	n.d. ^c^	n.d. ^c^	n.d. ^c^	n.d. ^c^	***
Sinapic acid hexoside 1	n.d. ^d^	7 ± 1 ^c^	n.d. ^d^	19 ± 1 ^b^	n.d. ^d^	35 ± 3 ^a^	n.d. ^d^	n.d. ^d^	n.d. ^d^	n.d. ^d^	***
Caffeoyl-hexoside 1	n.d. ^c^	8 ± 1 ^a^	n.d. ^c^	2.8 ± 0.4 ^b^	n.d. ^c^	3.1 ± 0.3 ^b^	n.d. ^c^	1.4 ± 0.1 ^b^	n.d. ^c^	1.4 ± 0.2 ^b^	***
Caffeoyl-hexoside 2	n.d. ^c^	0.6 ± 0.1 ^b^	n.d. ^c^	2.0 ± 0.0 ^a^	n.d. ^c^	2.6 ± 0.1 ^a^	n.d. ^c^	n.d. ^c^	n.d. ^c^	n.d. ^c^	***
Xanthone derivatives											
Mangiferin	n.d. ^b^	0.10 ± 0.01 ^a^	n.d. ^b^	n.d. ^b^	n.d. ^b^	n.d. ^b^	n.d. ^b^	n.d. ^b^	n.d. ^b^	n.d. ^b^	***
Norathyriol	n.d. ^c^	n.d. ^c^	n.d. ^c^	0.07 ± 0.02 ^a^	n.d. ^c^	0.02 ± 0.01 ^b^	n.d. ^c^	0.02 ± 0.01 ^b^	n.d. ^c^	n.d. ^c^	***
Galloyl derivatives											
Mono-galloyl-glucose 1	n.d. ^e^	518 ± 16 ^a^	n.d. ^e^	384 ± 25 ^b^	n.d. ^e^	266 ± 42 ^c^	n.d. ^e^	19 ± 2 ^d^	n.d. ^e^	17 ± 5 ^d^	***
Mono-galloyl-glucose 2	n.d. ^c^	220 ± 34 ^a^	n.d. ^c^	242.5 ± 0.5 ^a^	n.d. ^c^	173 ± 32 ^b^	n.d. ^c^	n.d. ^c^	n.d. ^c^	n.d. ^c^	***
Total phenolic compounds	n.d. ^c^	4057 ± 310 ^a^	0.9 ± 0.1 ^g^	4095 ± 239 ^a^	0.4 ± 0.1 ^f^	4202 ± 364 ^a^	0.4 ± 0.1 ^f^	2286 ± 288 ^b^	n.d. ^c^	2043 ± 135 ^b^	***

Different letters within each row indicate significant difference among samples. *** *p*-value < 0.001. n.d.: not detected.

## Abbreviations

UHPLC-HRMS	ultra-high-performance liquid chromatography coupled to high resolution mass spectrometry
FA	formic acid
FW	fresh Weight
BOD	Before Oral Digestion
AOD	After Oral Digestion
AGD	After Gastric Digestion
AID	After Intestinal Digestion
RT	retention time
[M-H]^−^ Exp	experimental exact mass
Δ	mass error
OFN	oxygen free nitrogen
BMIs	body mass index
n.d.	not detected
